# The Ethanol Extract of Licorice* (Glycyrrhiza uralensis)* Protects against Triptolide-Induced Oxidative Stress through Activation of Nrf2

**DOI:** 10.1155/2017/2752389

**Published:** 2017-09-26

**Authors:** Ling-Juan Cao, Zhen-Yan Hou, Huan-De Li, Bi-Kui Zhang, Ping-Fei Fang, Da-Xiong Xiang, Zhi-Hua Li, Hui Gong, Yang Deng, Yan-Xia Ma, Huai-Bo Tang, Miao Yan

**Affiliations:** ^1^Department of Pharmacy, The Second Xiangya Hospital, Central South University, Changsha 410011, China; ^2^Institute of Clinical Pharmacy, Central South University, Changsha 410011, China; ^3^School of Pharmaceutical Sciences, Central South University, Changsha 410013, China; ^4^School of Pharmacy, Hunan University of Chinese Medicine, Changsha 410028, China; ^5^Department of Pharmacy, Chemistry College, Xiangtan University, Xiangtan 411105, China

## Abstract

To investigate the potential role of nuclear factor erythroid 2-related factor 2 (Nrf2) in licorice ethanol extract (LEE) against triptolide- (TP-) induced hepatotoxicity, HepG2 cells were exposed to LEE (30, 60, and 90 mg·L^−1^) for 12 h and then treated with TP (50 nM) for 24 h. Besides, an acute liver injury model was established in ICR mice by a single dose of TP (1.0 mg·kg^−1^, i.p.). Relevant oxidant and antioxidant mediators were analyzed. TP led to an obvious oxidative stress as evidenced by increasing levels of ROS and decreasing GSH contents in HepG2 cells.* In vitro* results were likely to hold true in* in vivo* experiments. LEE protected against TP-induced oxidative stress in both* in vitro* and* in vivo *conditions. Furthermore, the decreased level of Nrf2 in the TP-treated group was observed. The mRNA levels of downstream genes decreased as well in ICR mice liver, whereas they increased in HepG2 cells. In contrast, LEE pretreatment significantly increased the level of Nrf2 and its downstream genes. LEE protects against TP-induced oxidative stress partly via the activation of Nrf2 pathway.

## 1. Introduction

Traditional Chinese medicine has focused on herb-herb interactions to achieve great efficiency, and the combination therapies have been validated and show potential clinical benefits [1]. Licorice, derived from the roots of* Glycyrrhiza uralensis*, is one of the most widely used herbal medicines, as it appears in more than half of traditional Chinese medicine prescriptions [1]. It contains various bioactive secondary metabolites including free phenolic compounds (flavonoids, coumarins, and so on), flavonoid glycosides, and triterpenoid saponins [2, 3]. It has antioxidation and toxicity countering effects, which could be described as detoxifying [2].* Tripterygium Wilfordii* Hook f. (TWHF) is a well-known herbal medicine that is widely used to treat various diseases, including rheumatoid arthritis, nephritic syndrome, and lupus [4]. Triptolide (TP), commonly used clinically, is a major active ingredient isolated from TWHF. However, its use is limited due to severe toxicities especially hepatotoxicity [4] and much evidence suggests that oxidative stress is a hallmark of TP-induced hepatotoxicity [5].

It is suggested that the activation of nuclear factor erythroid 2-related factor 2 (Nrf2) could protect against triptolide-induced hepatotoxicity [6]. Nrf2 is an emerging regulator of cellular resistance to oxidative stress [7]. Under the physiological condition, Nrf2 is presented in the cytoplasm binding to Kelch-like ECH-associated protein 1 (Keap1). Under the stress condition, Nrf2 dissociates from Keap1 and translocates into the nucleus, enhancing the expression of its downstream genes [7, 8]. Nrf2 pathway is considered as an important endogenous antioxidant pathway and increasing numbers of studies have suggested the potential role of Nrf2 as a therapeutic target to prevent liver injury caused by oxidative stress [9, 10].

Researches on combining administration of licorice and TWHF/TP have attracted more and more attentions. Effects of licorice on enhancing efficacy and reducing toxicity of TWHF/TP have been demonstrated in a rat collagen-induced arthritis model [11] as well as in the clinical application [12]. However, most of the researches focused only on pharmacodynamics and drug-drug interactions between TP and licorice [11–13]. Details of the hepatoprotection mechanism remain to be elucidated. We have previously reported that licorice ethanol extract (LEE) and its major representative active constituents, including liquiritin, liquiritigenin, isoliquiritigenin, and glycyrrhetinic acid, may intervene the Nrf2 pathway to induce its target genes, and this indicates a novel mechanism to lower drug toxicity and exert an antioxidation effect [14, 15]. Based on the observation, the current study aims to investigate the protective effect of LEE on TP-induced oxidative stress and the role of Nrf2 pathway using* in vitro* cells and* in vivo* mice models.

## 2. Materials and Methods

### 2.1. Chemicals and Reagents

**Figure 1:**
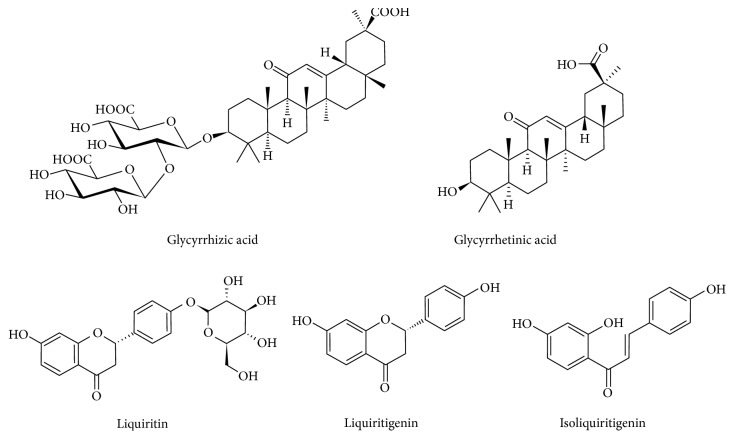
Chemical structures of five representative active compounds in licorice (glycyrrhizic acid, glycyrrhetinic acid, liquiritin, liquiritigenin, and isoliquiritigenin).

The dried roots of Glycyrrhiza uralensis were authenticated by Professor Shao Liu (School of Pharmaceutical Sciences, Central South University, Changsha, China). The voucher specimens were deposited in the Herbarium, School of Pharmaceutical Sciences, Central South University, Changsha, China. The purity of TP was more than 98.0% (HPLC), and the extraction, separation, and refining were finished in OnRoad Biotechnology Co., Ltd. (Changsha, China) from TWHF. Glycyrrhizic acid, liquiritin, liquiritigenin, and isoliquiritigenin were obtained from Chengdu Preferred Biological Technology Co. Ltd. (Chengdu, China), and the glycyrrhetinic acid was purchased from the National Institute for Food and Drug Control (Beijing, China). Purity of all the standards was above 98% by HPLC analysis. The chemical structures of them are shown in Figure 1. Methanol and acetonitrile (HPLC grade) were from ACS Company (Poole, UK). Formic acid (HPLC grade) was from ROE scientific INC (Network USA). Ultrapure water was deionized and purified by a MilliQ system (Millipore, Bedford, MA, USA). Tert-butylhydroquinone (tBHQ), dimethyl sulfoxide (DMSO), and methyl thiazolyl tetrazolium (MTT) were purchased from Sigma-Aldrich (St. Louis, MO, USA). Silymarin (the purity was 83.3%) was purchased from Shengtianyu Biotechnology Co. Ltd. (Wuhan, China). Paraformaldehyde was purchased from Sinopharm Chemical Reagent Co. Ltd. (Wuhan, China). Anti-Nrf2 antibodies were purchased from Santa Cruz Biotechnology (CA, USA). Other chemicals were of analytical grade from commercial suppliers.

### 2.2. Preparation and HPLC-MS/MS Analysis of LEE

The preparation of LEE was carried out in a controlled manner, as described elsewhere [3], in OnRoad Biotechnology Co. Ltd. (Changsha, China). The obtained LEE was analyzed through an LC-20A HPLC system (SHIMADZU, Kyoto, Japan), coupled with a 4000 triple-quadrupole mass spectrometer (AB SCIEX, Framingham, MA, USA). The Ultimate AQ-C18 (3.0 × 100 mm, 3.0 *μ*m) column and the Guard Cartridge System, C18 (4.0 × 2.0 mm) precolumn were used at the temperature of 40°C. The mobile phase solutions used for gradient separation were A, 0.1% formic acid in water and B, acetonitrile. A gradient elution program, at ambient temperature and a flow rate of 0.6 mL/min, was set as follows: 0.01 min, 98% A; 2 min, 92% A; 30 min, 75% A; 32 min, 70% A; 50 min, 55% A; 53 min, 15% A; 60 min, 15% A; 61 min, 92% A; 67 min, 98% A. Then the last mobile phase gradient was held till 75 min. The detection wavelengths were 256 nm and 330 nm. Compounds were detected by MS/MS with electrospray ionization (ESI) probe operated with enhance product ion (EPI) mode. Mass spectra were recorded at *m*/*z* 100–1000.

### 2.3. Cells Culture

Human hepatocarcinoma cell line HepG2 obtained from Xiangya Cell Bank (Changsha, China) was cultured in Dulbecco's modified Eagle's medium (Hyclone, Logan, USA) supplemented with 10% (v/v) fetal bovine serum (Sijiqing, Hangzhou, China) and antibiotics (50 U/mL of penicillin and 50 mg/mL streptomycin). Cells were grown in a 37°C incubator with 5% CO_2_.

### 2.4. MTT Assay

The cell viability was determined by MTT assay. Briefly, cells (2 × 10^4^/well) were plated in a 24-well plate for 24 h and preincubated with LEE, TP, and LEE + TP, respectively. At the end of incubation, MTT solution was added and further incubated for 3–5 h, after which MTT was removed and the formazan product formed was dissolved in DMSO. After shaking for 10 min, each sample was transferred to a 96-well microtiter plate and the absorbance was recorded at 490 nm.

### 2.5. Determination of Intracellular ROS and GSH

The fluorescent probe DCFH-DA was used to determine the level of reactive oxygen species (ROS). HepG2 cells (5 × 10^3^/well) were seeded in a black 96-well and then exposed to LEE (30, 60, and 90 mg·L^−1^) for 12 h, after which the complete media were removed and replaced with medium containing TP (50 nM) for 24 h. Then cells were incubated with DCFH-DA (10 *μ*M) for 20 min at 37°C. The fluorescent intensity was measured at an excitation wavelength of 488 nm and emission wavelength of 525 nm. For measurement of glutathione (GSH), HepG2 cells (6 × 10^5^/well) were exposed to the tested drugs as described above, after which the cells were harvested and lysed by ultrasonication. Following centrifugation at 3.5 × 10^3^ ×g for 10 min at 4°C, the supernatant was maintained on ice until assayed by GSH detection kit (Jiancheng Bioengineering Institute, Nanjing, China) according to manufacturer's instructions.

### 2.6. Animals and Experimental Protocol

All animal use procedures were conducted according to the Regulations of Experimental Animal Administration issued by the State Committee of Science and Technology of China, with the approval of the Ethics Committee in the Experimental Animal Center of the Second Xiangya Hospital. Six- to eight-week-old male ICR mice were purchased from Hunan SJA Laboratory Animal Co. Ltd. (Changsha, China). All mice were housed at 22–25°C and humidity 50–60% with a 12 h light-dark cycle and had free access to food and water.

Mice were randomly divided into seven groups (*n* = 6–8): (1) control, (2) TP (1.0 mg·kg^−1^), (3) TP + LEE (75 mg·kg^−1^), (4) TP + LEE (150 mg·kg^−1^), (5) TP + LEE (300 mg·kg^−1^), (6) TP + silymarin (200 mg·kg^−1^), and (7) high-dose of LEE (300 mg·kg^−1^). Silymarin, a commonly used liver protective agent, was used as positive control. Mice received either LEE or silymarin orally once daily for 7 days consecutively. One hour after the final treatment, mice were treated with TP (1.0 mg·kg^−1^, i.p.). The groups of control animals were given the corresponding vehicles. Six hours after the administration of TP, mice were given LEE or silymarin again. In all treated groups, mice were anesthetized for 24 h after TP injection.

### 2.7. Biochemical Assays

Blood samples were collected and serum was obtained for determining liver function by measuring alanine aminotransferase (ALT), aspartate aminotransferase (AST) (Wako, Osaka, Japan), alkaline phosphatase (ALP), and lactate dehydrogenase (LDH) (Medicalsystem, Ningbo, China) levels using commercially available enzymatic assay kits.

### 2.8. Antioxidant Enzymes

The extent of oxidative stress was estimated in liver homogenates by measuring activities of superoxide dismutase (SOD), catalase (CAT), glutathione peroxidase (GPx), and contents of GSH and malondialdehyde (MDA) using commercial kits (Jiancheng Bioengineering Institute, Nanjing, China) according to the manufacturer's instructions.

### 2.9. Tissue Processing and Staining

Liver samples taken from animals were immersed in a 4% (w/v) paraformaldehyde solution for 24 h. After fixation, the specimens were processed through graded alcohols, cleared in turpentine (substitute for xylene), and embedded in paraffin. Then the paraffin blocks were cut and stained with hematoxylin and eosin for morphological evaluation.

### 2.10. Western Blot

After treating with the tested drugs, liver samples were lysed with RIPA buffer (CW biotech, Beijing, China). Nuclear extracts were prepared with a Nuclear Extract Kit (Thermo, USA). Equivalent amounts of protein were separated by 10% SDS-PAGE and transferred to PVDF membranes. After being blocked in 5% nonfat milk in TBST for 1 h at room temperature, the membranes were incubated with the primary antibodies at 4°C overnight. Subsequently, the immunoblots were incubated with a secondary antibody at room temperature. The membranes were developed using an electrochemiluminescence (ECL) kit (Advansta, USA).

### 2.11. Quantitative Real-Time PCR Analysis

Total RNA was extracted with Trizol reagent (Invitrogen). Expressions of mRNA were quantified through real-time PCR technique, all with *β*-actin as control. PCR were performed using a SYBR Premix Ex Taq (Takara, Japan) and conducted with the ABI Prism 7900HT (Applied Biosystems, USA). Primers were shown in Table 1.

### 2.12. Statistical Analysis

Results from the experiments were reported as mean ± standard deviation (SD) calculated with SPSS 19.0. All data were analyzed by one-way ANOVA, followed by Tukey's test. Statistical significance was accepted at a *P* value less than 0.05.

## 3. Results

### 3.1. Representative Active Components of Licorice by HPLC-MS/MS

**Figure 2:**
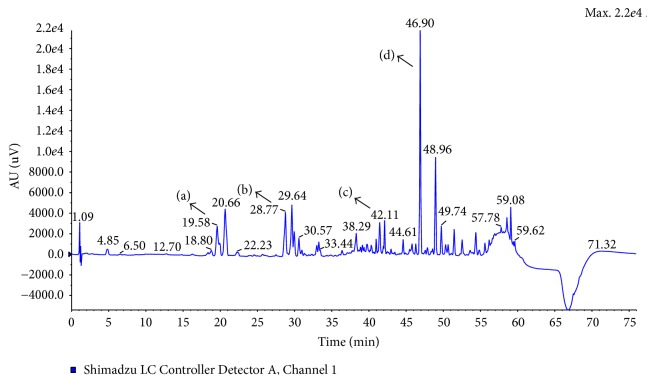
Separation of major chemical constituents of licorice ethanol extracts by HPLC-MS/MS. Peak identities and contents are (a) liquiritin (2.9%), (b) liquiritigenin (5.3%), (c) isoliquiritigenin (2.2%), and (d) glycyrrhizic acid (10.8%), based on accurate mass, retention time, and peak areas.

As shown in Figure 2, four major components of licorice were identified and well separated. Contents of them were also presented as follows: liquiritin, 2.9%; liquiritigenin, 5.3%; isoliquiritigenin, 2.2%; and glycyrrhizic acid, 10.8%. Data of glycyrrhetinic acid was not shown for its low abundance.

### 3.2. LEE had a Protective Effect on Cell Viability

**Figure 3:**
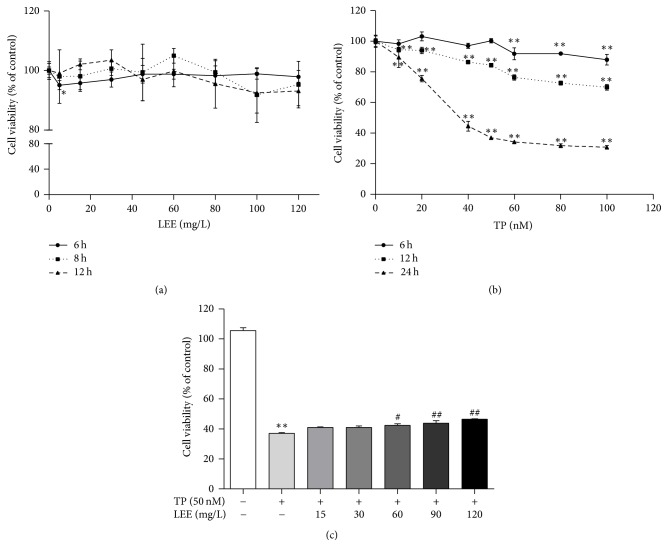
Cytotoxicity of LEE (a), TP (b), and LEE + TP (c) in HepG2 cells (*n* = 3). ^*∗∗*^*P* < 0.01 versus control; ^#^*P* < 0.05, ^##^*P* < 0.01 versus TP.

HepG2 cells were employed for investigating the potential toxicity of drugs. As shown in Figure 3(a), LEE-treated group exhibited no obvious cytotoxicity. However, TP decreased the cell viability dose- and time-dependently (Figure 3(b)). TP 50 nM was used in subsequent experiments, which was close to the half maximal inhibitory concentration (IC50). Next, Figure 3(c) showed that 12 h pretreatment with LEE protected HepG2 cells from subsequent TP-induced cell damage dose-dependently, increasing cell viability by up to 9.5% at the concentration of 120 mg·L^−1^.

### 3.3. Oxidative Stress and Antioxidative Activity in HepG2 Cells

**Figure 4:**
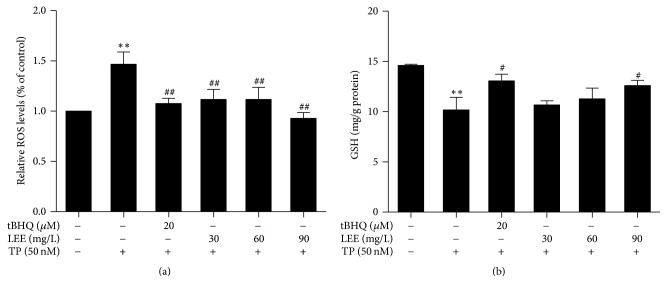
The contents of intracellular ROS (a) and GSH (b) in HepG2 cells were measured (*n* = 3). ^*∗∗*^*P* < 0.01 versus control; ^#^*P* < 0.05, ^##^*P* < 0.01 versus TP.

To determine the intracellular level of oxidative stress, contents of ROS were measured and tBHQ, a classical activator of Nrf2, was used as a positive control. The results revealed that TP exposure led to a significant increase in ROS levels (up by 50%) when compared to the control. Pretreatment with LEE for 12 h significantly decreased ROS levels compared with the TP-treated group (Figure 4(a)).

GSH levels were measured as an index of the nonenzymatic defense system in HepG2 cells. The results showed that TP significantly decreased GSH levels and LEE prevented the decrease although the difference was not statistically significant at 30 mg/L and 60 mg/L (Figure 4(b)).

### 3.4. Effects of LEE and TP on the Protein Levels of Nrf2 in HepG2 Cells

**Figure 5:**
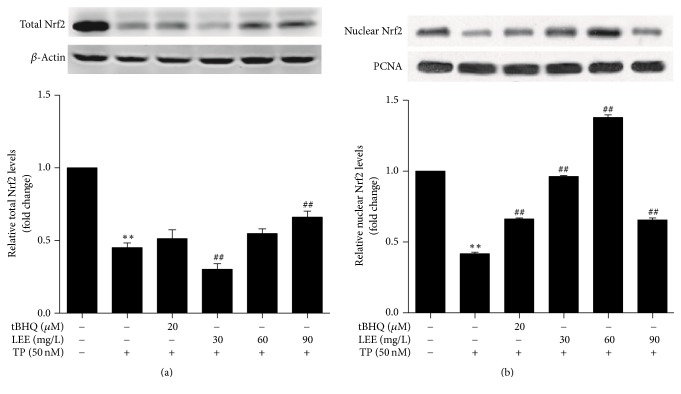
The protein levels of total and nuclear Nrf2 in HepG2 cells after treated with test drugs (*n* = 3). ^*∗∗*^*P* < 0.01 versus control; ^##^*P* < 0.01 versus TP.

As shown in Figure 5, TP pronouncedly decreased the total and nuclear protein levels of Nrf2. Pretreatment with different concentrations of LEE (60, 90 mg·L^−1^) for 12 h increased the total Nrf2 level compared to TP group. However, low-dose of LEE (30 mg·L^−1^) significantly decreased the total Nrf2 levels, which may be explained by that some ingredients acting as Nrf2 inhibitors existed and worked at lower concentrations. Furthermore, the nuclear Nrf2 levels were significantly upregulated following LEE (30, 60 mg·L^−1^) pretreatment. However, no further increase in the protein levels was observed at higher concentration of LEE (90 mg·L^−1^).

### 3.5. The mRNA Levels of Nrf2-Downstream Genes in HepG2 Cells

**Figure 6:**
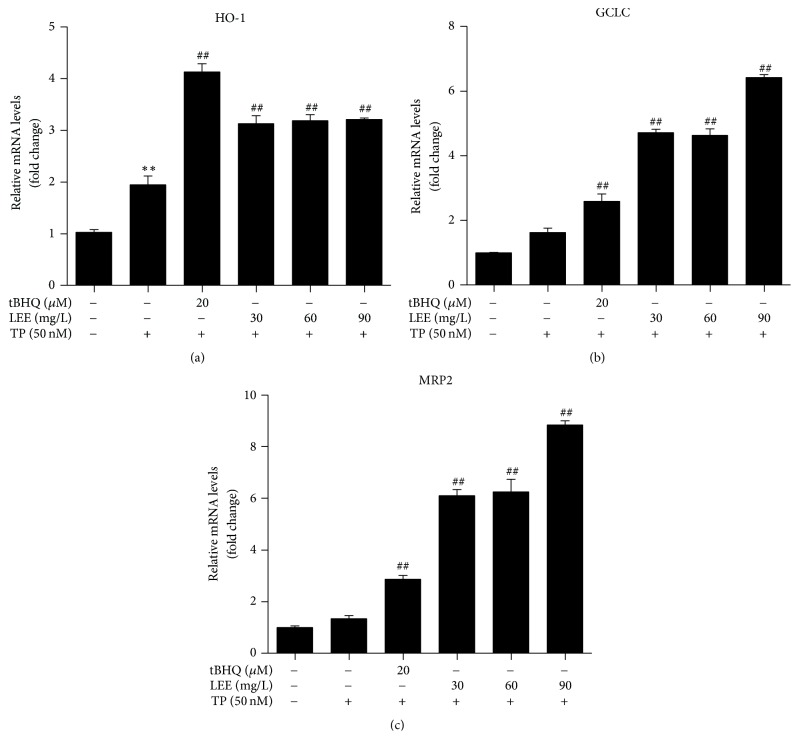
The mRNA levels of HO-1, GCLC, and MRP2 in HepG2 cells (*n* = 3). ^*∗∗*^*P* < 0.01 versus control; ^##^*P* < 0.01 versus TP.

As seen in Figure 6, TP treatment increased the mRNA levels of HO-1, GCLC, and MRP2. Comparatively, LEE gave a further significant increase.

### 3.6. Protective Effect of LEE on TP-Induced Liver Injury in ICR Mice

An animal model of acute liver injury induced by TP was established. As shown in Table 2, serum activities of ALT, AST, ALP, and LDH in the TP-treated group increased by 10.9-, 8.4-, 1.3-, and 3.9-fold, respectively, when compared with those in the control group. However, administration of LEE reduced the increase of ALT, AST, and LDH. Silymarin (200 mg·kg^−1^), a well-characterized hepatoprotective compound, reduced the increase as well. Additionally, high-dose of LEE alone did not give rise to significant changes when compared to control.

**Figure 7:**
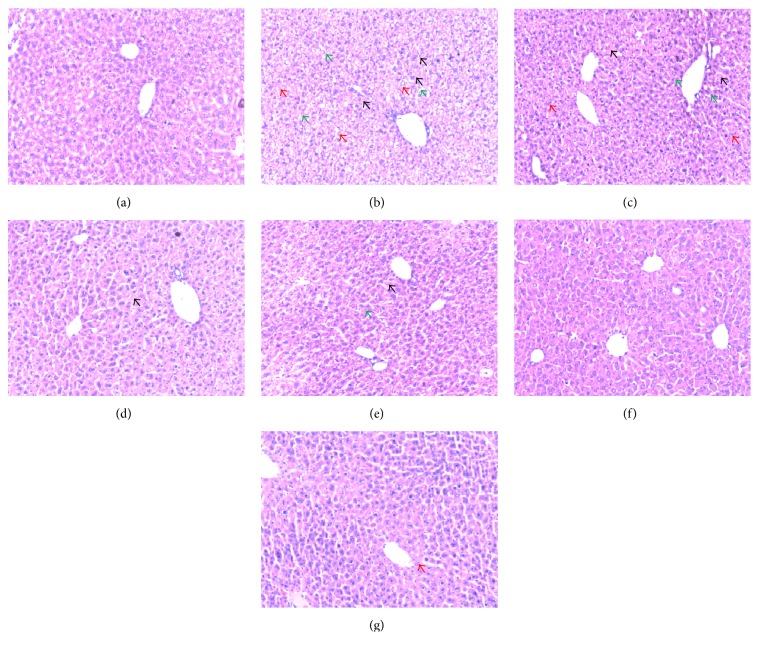
Histopathological analysis of the livers of ICR mice (magnification ×100). (a) Control; (b) TP (1.0 mg·kg^−1^); (c) TP + LEE (75 mg·kg^−1^); (d) TP + LEE (150 mg·kg^−1^); (e) TP + LEE (300 mg·kg^−1^); (f) TP + silymarin (200 mg·kg^−1^); (g) LEE (300 mg·kg^−1^). Red arrows indicated hepatocellular hydropic degeneration, black arrows indicated necrosis, and green arrows indicated inflammatory cells infiltration.

Histopathological analysis showed that, compared with the control group (Figure 7(a)), liver sections of the TP group (Figure 7(b)) showed a derangement of the hepatic cord and large areas of hydropic degeneration. Nucleolysis and further hepatic parenchymal necrosis and some inflammatory cells infiltration can also be seen in the TP group, but not in the positive control group (Figure 7(f)). The incidence and severity of histopathological lesions were significantly decreased in the LEE + TP group, showing that smaller areas of hepatocyte degeneration, necrosis, and inflammatory cell infiltration were seen in the 75 mg·kg^−1^ LEE + TP group (Figure 7(c)), and the histopathological changes were much slighter in the 150 mg·kg^−1^ LEE + TP group (Figure 7(d)) and 300 mg·kg^−1^ LEE + TP group (Figure 7(e)). In the 300 mg·kg^−1^ LEE control group (Figure 7(f)), slight hydropic degeneration can be seen near the central vein.

### 3.7. Effect of LEE on TP-Induced Oxidative Stress

Oxidative stress was quantified through measuring activities of GPx, SOD, and CAT and contents of GSH and MDA in liver tissue homogenates (Table 3). Significant reduction in the activities of GPx, SOD, CAT, and GSH contents was found in TP-treated group compared to control, and MDA content showed a tendency of increase. LEE treatment increased the activities of GPx, SOD, CAT, and GSH contents in varying degrees, whereas the content of MDA decreased. Moreover, silymarin also decreased TP-induced oxidative stress as indicated by the increased activities of GPx, SOD, CAT, and GSH levels and the decreased content of MDA. Additionally, high-dose of LEE (300 mg·kg^−1^) treatment alone did not alter the levels of GSH and MDA and the activities of SOD and CAT except GPx.

### 3.8. Effect of LEE on Protein Levels of Nrf2 in ICR Mice Livers

**Figure 8:**
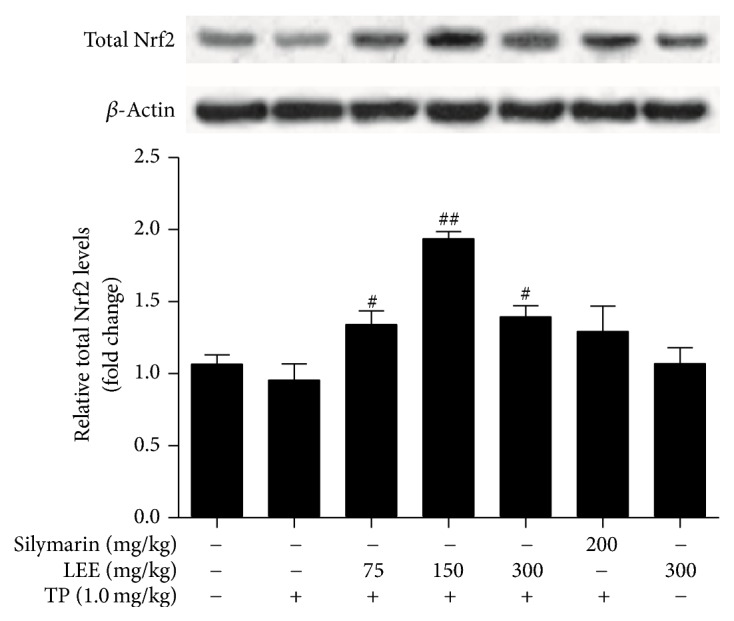
The protein levels of Nrf2 in the livers of ICR mice (*n* = 5). ^#^*P* < 0.05, ^##^*P* < 0.01 versus TP.

As presented in Figure 8, TP-treated mice showed a slighter decline level of total Nrf2 compared with control. And the protein levels of Nrf2 were significantly upregulated following LEE treatment.

### 3.9. Effect of LEE on mRNA Levels of Nrf2-Downstream Genes in ICR Mice Livers

**Figure 9:**
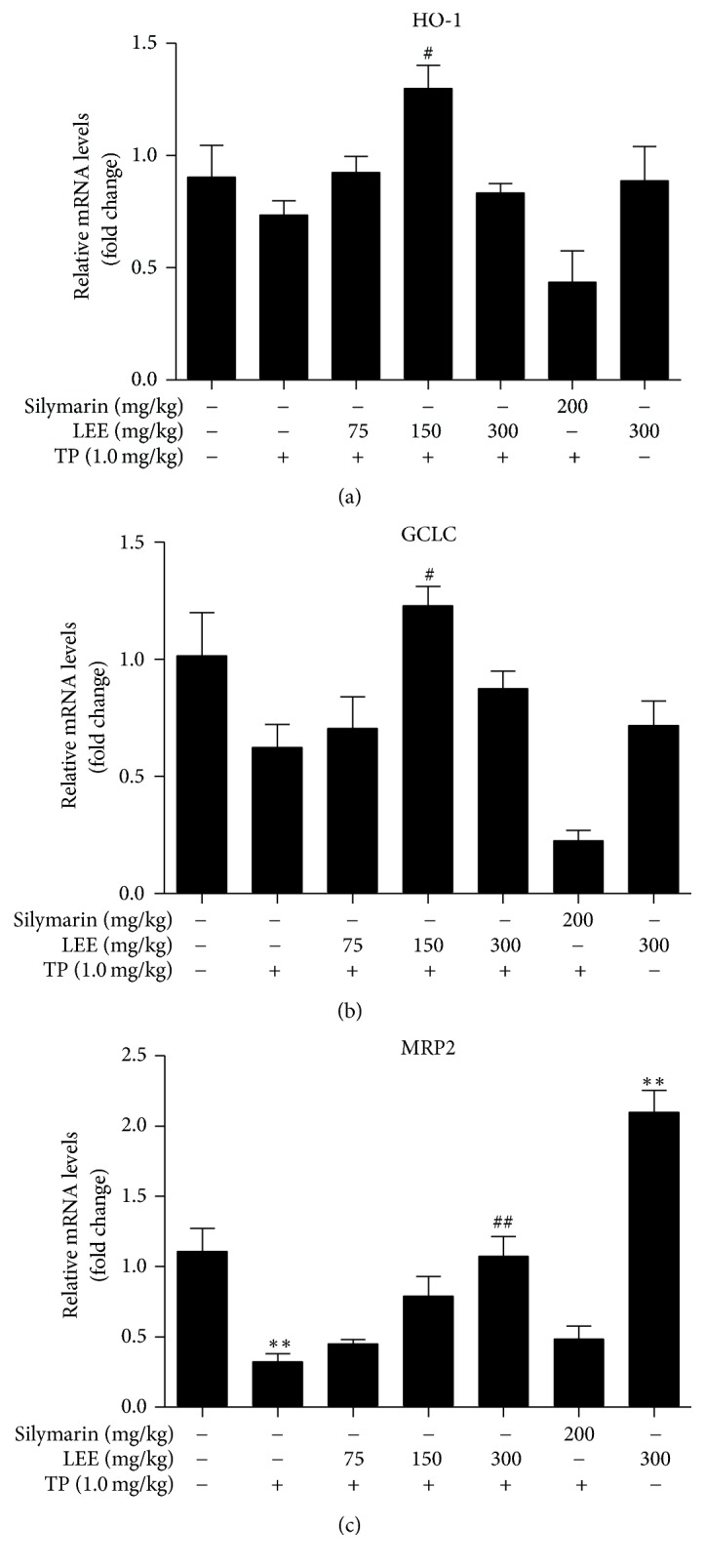
The mRNA levels of Nrf2-downstream genes in the livers of ICR mice (*n* = 5). ^*∗∗*^*P* < 0.01 versus control; ^#^*P* < 0.05, ^##^*P* < 0.01 versus TP.

To further confirm whether Nrf2-downstream genes are involved, mRNA levels of HO-1, GCLC, and MRP2 were analyzed (Figure 9). The results showed that TP caused a decrease in the mRNA levels which may be related to the protein levels of Nrf2 as shown in Figure 6. Additionally, the mRNA levels were increased in the LEE pretreated groups compared with TP group. These results indicated that activation of Nrf2, at least partly, is involved in the protective effect of LEE on TP-induced ICR mice liver injury.

## 4. Discussion

Licorice is widely used for the treatment of acute and chronic liver injury [16]. Several studies have shown that its major active components possess the liver protective function [1]. TP is a natural reactive electrophile containing three epoxide groups, which are linked to its toxicity via their ability to covalently bind to cellular macromolecules [17]. In the current study, oxidative stress caused by TP in HepG2 was confirmed through increasing levels of ROS and decreasing GSH contents, agreeing with other studies [6]. Nrf2 pathway may be involved in LEE suppressing TP-induced oxidative stress. Additionally, an animal model established in ICR mice was used to evaluate the protective role of LEE* in vivo*. Increased serum levels of AST, ALT, ALP, and LDH caused by TP have been attributed to the damaged structural integrity of the liver [18]. In addition, decreased levels of GSH, GPx, SOD, and CAT were observed in the TP group. Moreover, LEE effectively protected ICR mice against TP-induced liver injury. There was also an earlier study in rats that demonstrated a lowering effect of LEE on AST, ALT, and ALP levels in liver [19]. Additionally, these results were consistent with clinical researches [12] which demonstrated the toxicity attenuation and efficacy potentiation effect of licorice on the treatment of rheumatoid arthritis with TWHF.

Nrf2 is an important antioxidant transcription factor protecting against oxidative stress. This study indicated that the protective role of Nrf2 attributed partly to its involvement in coordinated inductions of antioxidative enzymes and drug transporters [20]. HO-1 functions as a rate-limiting enzyme in the breakdown of the prooxidant heme into carbon monoxide, free iron, and bilirubin [21]. It plays a key role in cellular defense mechanism against oxidative stress [22]. GCLC is the catalytic subunit of GCL, the enzyme that catalyzes the rate-limiting step in the biosynthesis of GSH [23]. MRP2, an efflux transporter participates in excretion of chemicals into bile, especially glutathione-, glucuronide-, and sulfate-conjugated metabolites [24]. In the current study, it is worthy noticing that the protein level of Nrf2 in the TP group decreased both* in vitro* and* in vivo*. Consequently, this may result in lower induction of antioxidant defenses, thus suffering aggravated damage [25]. Interestingly, though LEE increased Nrf2 target gene expressions such as GCLC and MRP2 in a dose dependent manner, nuclear Nrf2 was not. The reason may be that in addition to Nrf2, GCLC is also regulated by Nrf1 and pregnane X receptor (PXR); PXR is highly expressed in the liver and regulates the transcriptional expression of drug metabolizing enzymes [26, 27]. Besides, MRP2 is also regulated by numerous nuclear factors such as FXR, CAR, and PXR [28–30]. Hence, though the induction of Nrf2 by LEE contributes to the downstream induction of GCLC and MRP2, the regulation of GCLC and MRP2 by Nrf2 is partial but important.

Many studies including ours have discovered the activation effect of licorice and its active ingredients on Nrf2 pathway by exerting antioxidative and hepatoprotective functions [14]. Isoliquiritigenin, a flavonoid extracted from licorice, exhibited a suppressive effect on lipopolysaccharide-induced inflammatory responses which may be associated with inhibiting the Keap1, increasing Nrf2 translocation, and inducing the expression of target genes [31]. Another constituent glycyrrhetinic acid protected against CCL4-induced mice chronic liver fibrosis involving upregulating of Nrf2 [32]. Moreover, the retrochalcone licochalcone A could activate Nrf2* in vitro* and contribute to LEE-induced lowered cutaneous oxidative stress* in vivo* [33]. In addition, glycycoumarin might ameliorate alcohol-induced hepatotoxicity via activation of Nrf2 and autophagy [34, 35]. In summary, the activation of Nrf2 by licorice may be the aggregate effect of a wide variety of constituents. Though proper dose of licorice is cytoprotective by showing its antioxidant activity, it has been proved that overdose of licorice or its active constituents may result in compensatory redox imbalance and reductive stress [36]. As shown in Figure 8, compared with the TP injury group, combined treatment with LEE and TP did not show a concentration dependent induction of Nrf2 expressions, indicating that the maximum dose (300 mg/kg) of LEE may have a reductive effect on livers, so Nrf2 was decreased to adapt to this state when LEE displayed prooxidant activities. That is the reason why 300 mg/kg LEE alone did not activate the Nrf2* in vivo*. But when TP was administered, ROS was largely produced and cells were in an oxidant state, which made LEE promote the production of Nrf2 and the release of it from its inhibitory interaction with Keap1 and reduce the degradation of Nrf2. That is the reason why LEE could activate the Nrf2 pathway when combined with TP* in vivo*.

Collectively, the current study was the first to demonstrate that the hepatoprotection role of LEE against TP-induced oxidative stress and liver injury was possibly related to its ability to activate Nrf2 pathway* in vitro* and* in vivo*. This study suggested that licorice might be candidates for the prevention of drug-induced hepatotoxicity and Nrf2 pathway may present a new biological target. Therefore, it provided new and meaningful insights into protecting against drug-induced liver injuries.

## Figures and Tables

**Table 1:** Primers for the analysis of human and mice gene levels by qPCR.

	Gene	Forward (5′-3′)	Reverse (5′-3′)
Human	HO-1	GCCAGCAACAAAGTGCAAGA	AAGGACCCATCGGAGAAGC
	GCLC	GATGCTGTCTTGCAGGGAATG	AGCGAGCTCCGTGCTGTT
	MRP2	TGAGCAAGTTTGAAACGCACAT	AGCTCTTCTCCTGCCGTCTCT
	*β*-Actin	CGTGGACATCCGCAAAGAC	TCGTCATACTCCTGCTTGCTG

Mice	HO-1	GAGCAGAACCAGCCTGAACTA	GGTACAAGGAAGCCATCACCA
	GCLC	GCACGGCATCCTCCAGTTCCT	TCGGATGGTTGGGGTTTGTCC
	MRP2	ACTATCGCACACAGGCTGCAC	GGGACCCATATTGGACAGCA
	*β*-Actin	CGTGGACATCCGCAAAGAC	TCGTCATACTCCTGCTTGCTG

**Table 2:** Effect of LE on serum activities of AST, ALT, ALP, and LDH in mice treated with TP (*n* = 6), *x*  ±  SD.

Group	ALT/U·L^−1^	AST/U·L^−1^	ALP/U·L^−1^	LDH/U·L^−1^
Control	36.7 ± 12.8	101.1 ± 19.5	95.3 ± 34.1	692.8 ± 156.0
TP	400.3 ± 322.6^*∗∗*^	854.0 ± 596.4^*∗∗*^	120.0 ± 22.5	2702.9 ± 530.1^*∗∗*^
TP + LE 75	346.2 ± 192.7	570.0 ± 94.9	98.6 ± 14.3	2439.4 ± 304.7
TP + LE 150	63.0 ± 21.7^##^	178.7 ± 27.0^##^	93.7 ± 26.0	1234.4 ± 131.5^##^
TP + LE 300	85.1 ± 57.7^##^	182.0 ± 55.1^##^	91.7 ± 13.4	998.2 ± 203.8^##^
TP + silymarin	48.1 ± 37.0^##^	171.2 ± 77.9^##^	91.4 ± 16.4	737.4 ± 103.4^##^
LE 300	26.5 ± 4.4	127.3 ± 24.6	99.0 ± 6.4	732.4 ± 96.3

^*∗∗*^ *P* < 0.01 versus control; ^##^*P* < 0.01 versus TP.

**Table 3:** Effect of LE on the hepatic GSH, GPX, SOD, CAT, and MDA in mice treated with TP (*n* = 6), *x* ± SD.

Group	GSH (nmol/mg prot)	GPx (U/mg prot)	SOD (U/mg prot)	CAT (U/mg prot)	MDA (nmol/mg prot)
Control	39.70 ± 1.64	27.09 ± 1.11	4.02 ± 0.38	16.09 ± 0.67	2.41 ± 0.23
TP	31.37 ± 2.85^*∗∗*^	18.81 ± 0.46^*∗∗*^	2.51 ± 0.35^*∗∗*^	14.58 ± 0.17^*∗∗*^	3.40 ± 0.44
TP + LE 75	35.09 ± 2.84	22.97 ± 1.40^##^	2.98 ± 0.45	15.23 ± 0.41	2.84 ± 0.31
TP + LE 150	37.61 ± 2.91^#^	24.78 ± 1.32^##^	3.70 ± 0.47^#^	15.40 ± 0.37^#^	2.65 ± 0.52
TP + LE 300	36.82 ± 1.66	24.72 ± 1.46^##^	3.78 ± 0.20^#^	15.55 ± 0.36^##^	2.11 ± 0.10^##^
TP + Silymarin	38.52 ± 1.85^##^	24.72 ± 3.07^##^	4.09 ± 0.69^##^	15.35 ± 0.29	2.54 ± 0.36^#^
LE 300	37.69 ± 3.91	23.12 ± 1.20^*∗∗*^	3.56 ± 0.23	15.43 ± 0.23	2.47 ± 0.34

^*∗∗*^ *P* < 0.01 versus control; ^#^*P* < 0.05, ^##^*P* < 0.01 versus TP.
